# A Rome III survey of functional dyspepsia among the ethnic Malays in a primary care setting

**DOI:** 10.1186/1471-230X-13-84

**Published:** 2013-05-14

**Authors:** Yeong Yeh Lee, Noriza Wahab, Nazri Mustaffa, Norwati Daud, Noorhayati Mohd Noor, Juwita Shaaban, Andrew Seng Boon Chua

**Affiliations:** 1 Department of Medicine, School of Medical Sciences, Universiti Sains Malaysia, Kubang Kerian, Kelantan, Malaysia; 2 Department of Family Medicine, School of Medical Sciences, Universiti Sains Malaysia, Kubang Kerian, Kelantan, Malaysia; 3 Gastro Centre Ipoh, 31 Lebuh Raya Raman Ipoh, Ipoh Garden South, 31400, Ipoh, Perak, Malaysia

**Keywords:** Functional dyspepsia, Malays, Rome III, Females, Marriage, Psychosocial alarm

## Abstract

**Backgrounds:**

The study aimed to survey for FD in a primary care setting in a population known to have an extremely low prevalence of *Helicobacter pylori (H. pylori)* infection, with the hypothesis that in such a population, dyspepsia should have been relatively less common.

**Methods:**

The Rome III FD Diagnostic Questionnaire was translated into the Malay language and later tested for reliability. A prospective cross-sectional survey was then performed involving 160 Malay patients attending primary care clinic after informed consent. Patients positive for symptoms of FD were subjected to upper endoscopy and exclusion of *H. pylori* infection. Univariable and multivariable analyses were used to test for associated risk factors.

**Results:**

The back-translated questionnaire was similar to the original English version and was reliable (Cronbach Alpha-coefficient 0.85). Of the 160 surveyed subjects, 19 of them (11.9%) had symptoms of FD. With exclusion of erosive diseases (3/160 or 1.9%) from endoscopy, 16 subjects or 10% had FD. None of the 19 subjects were positive for *H. pylori* infection. Epigastric pain syndrome was present in 11/16 (68.8%) and the rest, overlap with postprandial distress syndrome. With multivariable analysis, a married status (OR = 8.1; 95% CI 1.0-36.5) and positive psychosocial alarm symptoms (OR = 3.8; 95% CI 1.0-14.0) were associated with FD. Of those married subjects, females were more likely to have FD and psychosocial symptoms than men (6.3% vs. 1.9%), *P* = 0.04.

**Conclusions:**

FD was more common than one had expected among Malays attending primary care clinic in an area with low prevalence of *H. pylori*.

## Background

In a recent study from a primary care setting in the Klang Valley of Peninsular Malaysia, uninvestigated dyspepsia was reported in approximately 1% (124/11037) of patients and 23.5% of them were tested positive for *Helicobacter pylori* (*H. pylori*) infection [1]. Dyspepsia related to *H. pylori* is prevalent within the Asia-Pacific region, however, in a survey, only 58% of primary care doctors had checked for *H. pylori* in their patients [2].

Previously uncommon in Asia, gastroesophageal reflux disease can account for 8.5 - 20% of causes of dyspepsia and it can overlap with functional dyspepsia (FD) in 2.3 - 8.4% of cases [3,4]. In a survey within the Asia-Pacific region, a diagnosis of FD was made in only 33% of primary care practitioners and only 40% of them were familiar with the term FD, probably even less had heard about Rome criteria [2].

In a population-based study involving 2000 subjects in a rural community (79% ethnic Malays) in Malaysia, uninvestigated dyspepsia (Rome II) was reported in 14.6%, with the ethnic Malays having a lower prevalence when compared to the Chinese (14.6% vs. 19.7%) [5], this being possibly a consequence of their lower susceptibility to *H. pylori* infection [6-8]. Data from the primary care were unfortunately scarce in Asia, which prompted our research.

The current observational study aimed to survey for FD in a primary care setting in a population known to have an extremely low prevalence of *H. pylori* infection, with the hypothesis that in such a population, dyspepsia should have been relatively less common than one would have expected.

## Methods

### Translation of Malay language Rome III FD Diagnostic questionnaire

The English version Rome III FD Diagnostic questionnaire was translated into the Malay language by 2 independent translators; one a bilingual physician (YYL) and the other, a qualified linguistic expert. The translators then met together with the research coordinator (JS) for reconciliation of differences as outlined in the guidelines issued by the Rome foundation [9]. The revised Malay translated version of the FD questionnaire was then back translated into English by another qualified local linguistic expert who then met with the research coordinator for reconciliation. Further revisions were made to the Malay version if needed during a subsequent reconciliation process involving two linguistic experts, two clinicians and the research coordinator. All revisions made to both forward and backward translations during the reconciliation process were carefully documented in a table showing the source language, target language version, comments and revisions made. The Malay translation was then tested for internal consistency, randomly, in 50 subjects who had a good command of the Malay language.

The Red Flag and the Psychosocial Alarm Questionnaires used in the current study were previously translated into the Malay language and had been validated for use in the local population. The intra-class correlation co-efficient (ICC) for the Red Flags and Psychosocial Alarm Questionnaires was 0962 and 0.994 respectively [10]. Briefly, the Red Flag Questionnaire includes history for the past three months of fever, weight loss, cancer in family members, blood mixed with stool, anaemia and change in bowel habit after age 50. The Psychosocial Alarm Questionnaire has 7 items designed to identify those patients with psychological “problems” and who may benefit from psychological evaluation. There are two questions for anxiety and depression of which the answers, “most or a lot of the time” identified the presence of these disorders with a mean Hospital Anxiety and Depression Scale (HADS) score of 13.3 and 9.0 respectively (compared with HADS mean score of 6.0 and 5.0 in those who answered “occasionally or not at all”) [11].

### Definitions and diagnosis of FD according to Rome III

According to the Rome III definition, FD is characterized by bothersome postprandial fullness, early satiation, epigastric pain and burning for the past 3 months with at least 1 day per week or more with onset of more than 6 months prior to diagnosis and no evidence of structural disease at endoscopy [11]. It is subdivided into two meal-related syndromes namely post-prandial distress syndrome (PDS) and epigastric pain syndrome (EPS). Briefly, PDS is characterized by bothersome post-prandial fullness after ordinary sized meals and or early satiation that prevent finishing a regular meal with frequency of at least several times per week. With EPS, there is intermittent pain or burning discomfort of at least moderate severity, localized to epigastrium and occurs for at least a day per week. The pain or burning discomfort often disappears completely in the same day, not relieved by defecation or flatulence and not fulfilling criteria for gallbladder or sphincter of Oddi disorders.

The Rome III FD questionnaire consists of 18 items with answers in ordinal scale and individual frequency thresholds. Item 3, 5 and 7 consist of questions on frequency of symptoms of post-prandial fullness, early satiation and epigastric pain or burning in the last 3 months with items 3, 5 and 8 for determination of symptom onset for 6 months or longer in a yes or no response. Items 3 and 5 are questions diagnostic for PDS and items 1, 2, 7, 9, 10 and 12 are questions that characterize EPS. Other items from 11 to 18 contain questions to exclude functional gallbladder and sphincter of Oddi disorders.

### Cross-sectional survey of FD

#### Study subjects

The translated questionnaires were subsequently used in a prospective cross-sectional survey for FD involving only ethnic Malays attending a primary care clinic within the university hospital of Universiti Sains Malaysia (USM). The hospital is located in the heart of Kota Bahru, the state capital of Kelantan, with an estimated population of 570,000 and 90% of its racial diverse population consists of ethnic Malays [12], all embracing the Islamic faith.

Consecutive Malay subjects (18 years old and above) presented at the primary care clinic were screened by the study investigator (NW) for inclusion into the study. Using a systematic 1:2 random sampling approach, those subjects randomized for screening were then called-in for an interview to determine for their study suitability. Subjects who were able to understand and complete the Malay translated questionnaires and also agreed for subsequent upper endoscopy examination were included. Those subjects with a known organic cause for their dyspeptic symptoms including previous peptic ulcer disease, previous endoscopy showing reflux disease and gastro-duodenal diseases, upper gastrointestinal tract cancers and psychiatric illnesses were excluded.

A total of 192 Malay subjects were initially randomized and screened for inclusion into the study. This sample size was calculated with a good power and to allow for multivariate analysis, the two proportion formula (for categorical variables) and the two means formula (for continuous variables) were used to derive the sample size calculated with the PS software. After considering 20% drop-out, a power of 80%, a precision of 0.05 and an estimated proportion of 11.8% [13], the calculated sample size was 192.

#### Study protocol

All enrolled subjects were given a questionnaire booklet to answer, usually within 20–30 minutes. The booklet contained Malay translated Rome III FD questionnaire, Red Flags and Psychosocial Alarm Questionnaires as well as socio-demographic questions including age, gender, educational background, occupation, individual income, number of children, smoking habits, drugs taken and past medical history. We have included the Red flags and Psychosocial Alarm Questionnaires in the current study since the association between red flags and psychological symptoms with FD is unknown in this population.

Only one trained interviewer (AW) was involved to maintain consistency in data collection and the interviewer was a clinician experienced with the Rome III questionnaires and Rome III diagnostic criteria. The body mass index or BMI (in Kg/m^2^) was determined in all study subjects, with height measured using a standard measuring tape (in cm) and weight with a calibrated weighing machine (in Kg) (Seca Deutschland, Hamburg, Germany). Those subjects who fulfil the Rome III criteria for FD were then referred for an upper endoscopy examination to rule out other possible upper gastrointestinal causes of their dyspeptic symptoms.

All upper endoscopy was performed by one experienced endoscopist (NM) who was blinded to the study; without knowing the diagnosis of FD beforehand in study subjects. All subjects were examined for *H. pylori* infection using CLO-test and histology with biopsies taken from at least two sites including antrum and body.

If there was further suspicion of any other gastrointestinal diseases and or red flags were positive then investigations were organized during subsequent follow-up at the gastroenterology clinic. Subjects with psychosocial alarm symptoms were then referred to psychiatrist if and when needed.

The study was approved by the Human Ethics Committee of USM.

### Data and statistical analysis

All data were recorded in mean, standard deviation (SD), frequency and percentage unless otherwise stated. Univariable logistic regression was used to test the association of independent variables (including age, sex, BMI, education level, occupational status, family income level, marital status, smoking status, red flags and psychosocial alarm features) with the presence or absence of FD, these are reported as crude odds ratio (OR) and 95% confidence interval (CI). Variables with statistical significance below 0.05 from univariable analysis were subsequently tested using the multivariable logistic regression analysis and reported as adjusted odds ratio, 95% CI and *P* value. All analyses were carried out using SPSS version 18.0 (SPSS Inc, IL, Chicago). A *P* value < 0.05 was considered statistically significant for all analyses, including non-parametric tests.

## Results

### Translation of the Rome III FD diagnostic questionnaire

During reconciliation of forward translation, item 3 and 4 that described on post-prandial fullness did not achieve an agreement between translators on the appropriate local term for “fullness”. The research coordinator made a decision on the final term after consulting with both translators, interviewing local patients and from results of pilot testing. Otherwise only minor adjustments were needed to item 12 and 14. No modifications were needed for back-translation into the English version. The final back-translated version was similar to the original English version. The translated questionnaire was approved by the translation committee of the Rome foundation. With pilot testing in 50 subjects, the Cronbach Alpha-coefficient for the translated FD questionnaire was 0.85.

### Upper endoscopic examination

Out of 192 screened subjects, 160 subjects were enrolled after informed consent with a response rate of 83.3%. The other 32 subjects either refused endoscopy or did not complete the questionnaire to allow analysis of data. Among the 160 surveyed subjects, 11.9% (19/160) fulfilled the Rome III criteria for FD. Out of these 19 subjects, upper endoscopy examination was reported as normal in 84.2% (16/19). Out of the three subjects with abnormal upper endoscopic examination (15.8%), two subjects had erosive esophagitis and one subject had erosive gastritis. None of the 19 subjects had evidence for *H. pylori* infection with CLO-test and or histology. Based on the findings of endoscopy and *H. pylori* testing, 10% (16/160) of surveyed subjects would be considered as having FD.

### Characteristics of FD and its meal-related subtypes

The mean (SD) age of the 160 surveyed subjects was 39.6 (14.3) years old. Subjects with FD were older than those without FD, with a trend towards statistical significance (*P* = 0.06) (Table 1). There was no difference between gender with and without FD (*P* = 0.7). Of the 16 subjects with confirmed FD, EPS accounted for 68.8% (11/16) with the rest, 31.2% (5/16) having PDS overlapped with EPS. Red flags were reported in 37.5% (6/16) of subjects with FD or 3.8% (6/160) of the total surveyed subjects (Table 1). Psychosocial alarm symptoms were present in 81.3% (13/16) of subjects with FD or 8.1% (13/160) of surveyed subjects (Table 1).

**Table 1:** Results of simple logistic regression analysis

**Parameters**	**FD n = 16**	**Not FD n = 144**	**Crude OR (95% CI)**	**P value**
Age^*^	46.2 (9.4)	38.8 (14.5)	1.04 (0.99, 1.08)	0.06
Body Mass Index^*^ (kg/m^2^)	28.1 (4.9)	25.2 (4.7)	1.13 (1.02, 1.26)	0.03^@^
Family income^*^ (RM)	2628.1 (2253.7)	2607.3 (2106.0)	1.00 (1.00,1.00)	1.0
Sex Male	6 (37.5)	62 (43.1)	1.00	0.7
Female	10 (62.5)	82 (56.9)	1.26 (0.44,3.65)	
Education level				0.09
Non-tertiary education	12 (75.0)	75 (52.1)	1.00	
Tertiary education	4 (25.0)	69 (47.9)	0.36 (0.11, 1.18)	
Occupational status				0.2
Not working	6 (37.5)	77 (53.5)	1.00	
Work full-time	10 (62.5)	67 (46.5)	1.92 (0.66, 5.55)	
Marital status				0.04^@^
Single or divorced	1 (6.3)	54 (37.5)	1.00	
Married	15 (93.8)	90 (62.5)	9.00 (1.16, 70.06)	
Smoking status				0.7
Non-smoker	13 (81.3)	122 (84.7)	1.00	
Current and ex-smoker	3 (18.8)	22 (15.3)	1.28 (0.34,4.86)	
Red flag symptoms				0.1
Symptom absent	10 (62.5)	116 (80.6)	1.00	
Symptom present	6 (37.5)	28 (19.4)	2.49 (0.83, 7.42)	
Psychosocial symptoms				0.03^@^
None	3 (18.8)	71 (49.3)	1.00	
Symptom present	13 (81.3)	73 (50.7)	4.22 (1.15,15.42)	

FD; functional dyspepsia, OR; odd ratio, CI; confidence interval, n; number of subjects, RM; Ringgit Malaysia.

All parameters in n (%) except those parameters marked with ^*^ in mean (standard deviation).

^@^significant on univariate analysis with P value <0.05.

### Univariable and multivariable analysis

With univariable analysis, a higher BMI, a positive married status and the presence of psychosocial alarm symptoms were associated with FD in the current study (*P* = 0.03, 0.04 and 0.03 respectively) (Table 1). Of these three factors, a married status (OR = 8.1) and the presence of psychosocial alarm symptoms (OR = 3.8) remained significant with multiple logistic regression analysis (Table 2).

**Table 2:** Results of multiple logistic regression analysis

**Factors**	***P*value**	**OR**	**95% CI for OR**
Positive marital status	0.05	8.08	1.03 – 36.51
Presence of psychosocial symptoms	0.05	3.76	1.01 – 13.99

Area under the receiver operating curve (ROC) = 0.75, Hosmer & Lemeshow test *P* value = 0.18, OR; adjusted odd ratio, CI; confidence interval.

### Further evaluations on marriage status, psychosocial symptoms and FD

Of those married subjects, females were more likely to have FD and psychosocial symptoms than men (6.3% vs. 1.9%, *P* = 0.04). None of the 30 unmarried females compared to 10 out of 62 married females had FD. Married females with FD were likely to report EPS rather than overlap between EPS and PDS (4.4% vs. 1.9%, *P* = 0.03). Based on the two questions on anxiety and depression from the Psychosocial Alarm Questionnaire, among the married females with FD; 1.3% or 2/160 subjects reported anxiety symptom “a lot of the time”, none reported depressive symptom “most or a lot of the time”, however, 4.4% or 7/160 subjects reported anxiety and depressive symptoms “occasionally”.

## Discussion

Cross-cultural translation of Rome III questionnaires is important to allow comparative studies between different ethnic groups but it can be challenging to retain the meaning and intent of the original instrument. In the current study, the Malay language translation of the English language version of the Rome III FD Diagnostic questionnaire was performed by linguistic experts in collaborations with clinicians, both familiar with local nuances and culture. The Malay-translated questionnaire, translated based on guidelines provided by the Rome foundation, was approved by the Rome III committee and is available with permission from its website [14]. Other than the symptom of “fullness” with local nuances, the back-translated version was comparable to the original English version. The translated questionnaire was tested for internal consistency and was found to be reliable, comparable to other previously reported studies [15,16]. Other detailed psychometric properties and validation were not evaluated for the current questionnaire since these properties had been previously evaluated in a similar Rome III Questionnaire [10].

A true prevalence of FD in the general population is difficult to determine, but available data indicate the prevalence could range between 11% and 29%, depending on whether the Rome criteria was used [17]. Being a more clinically relevant population to study, approximately 20% of primary care consulters report symptom of dyspepsia, which is largely functional in origin [18]. However, among the Asian population, with H. pylori infection being prevalent, a diagnosis of FD has been less straightforward, with significant overlap between the two [18].

Of those 160 surveyed Malay subjects attending primary care clinic, 19 patients or 11.9% fulfilled the Rome III symptom criteria for FD. Upper endoscopy at diagnosis was able to exclude three subjects (1.9%) having erosive diseases. Predominant symptoms of GERD, if present, effectively exclude FD according to the Rome III criteria. However, in actual practice, both conditions are not easily distinguished from each other if based on symptoms alone, since overlap exists [18]. None of the Malay subjects with FD had *H. pylori* infection as one would have expected from this population. With the exclusion of erosive diseases, by definition, 16 subjects or 10% were therefore classified as having FD. The north-eastern region of Peninsular Malaysia consists of ethnic Malays in 90% of its population and is very much rural and poor when compared to the West coast of the Peninsular. The prevalence of *H. pylori* infection is also exceptionally low in this region in the order of 4 - 5% and likewise organic dyspepsia [6-8,19-21]. The reported FD rate of 10% in the current study was therefore not as uncommon as one would have expected.

One striking finding in this population was the association between a positive marital status and FD. Of those subjects with a married status, females were more likely to have FD than men. A similar finding was reported by Moayyedi et al. in that 38% of married women reported more dyspepsia compared to 26.5% of single women [22]. The reasons for this association are unknown. Some studies have reported that marriage protects against depression but this effect could be greater for men than women [23,24]. Data suggest that the divorce trend since 1948 among the Malay women in Kelantan has remained high (approximately 80 in 100 marriages), the highest among the three Malay states of Kelantan, Kedah and Melaka in Malaysia [25]. It is also unique that in this region of the Peninsular, women are the breadwinner of the family rather than the men [26]. Due to above reasons, it is conceivable that women from this population might suffer more stress and anxiety and they are unlikely to report these to their medical practitioners [27]. As a result, functional and psychosocial symptoms were manifested masking the underlying psychosocial disturbance. In the current study, all the 10 married females with FD reported some psychosocial symptoms but only two of them reported significant anxiety symptom with the majority reported both anxiety and depressive symptoms only “occasionally”. Further studies are warranted to determine this association between functional gastrointestinal disorders with marriage and psychosocial symptoms.

Of the two meal-related syndromes, EPS was more commonly reported among the Malays than the overlap syndrome with PDS (68.8% vs. 31.2%). This was found to be similar to the Muslim population from Pakistan (EPS rate of 57%) [28], but this was in contrast to studies from Korea and Japan where PDS was reported to be more common than EPS (47% vs. 26% and 29.8% vs. 12.5% respectively) [29,30]. Studies have shown that PDS is often associated with IBS and FD-IBS overlap but compared to EPS, there is less effect on the quality of life [31]. It is unknown why EPS was more common in the current study but married Muslim females appeared to have a higher risk. It is interesting to note the similarity in meal-related syndrome between the Malays and the Muslim population from Pakistan, raising the question that whether a suppressed role of Muslim females in these communities may have contributed to their symptoms. In some studies, EPS was found to be more commonly associated with GERD overlapping with FD [31]. It is a limitation that the current study did not include assessment for GERD and IBS which might provide further information on the nature of overlap syndrome.

Red flags, the indicator for the presence of organic diseases, were reported in a third of Malay subjects with FD. With IBS, in the same Malay population, red flags were more common, being reported in approximately three-quarters of patients [32]. In both disorders, however, the diagnostic yield for organic diseases especially malignancy are extremely low, reiterating the underlying limitation of alarm symptoms being elicited in these conditions, a finding shared with other reports in Asia and elsewhere [33,34].

BMI appeared to be of less significance in FD compared to marital status and psychosocial dysfunction, but this might have been due to our research setting since subjects attending primary care clinic were relatively more ill. The results that subjects with FD were more likely obese, might have partly explained why EPS was more common in our population, since GERD is more commonly associated with both [35]. Further studies are needed to address this issue.

What is the relevance of our results to the management of dyspepsia among ethnic Malays in the primary care? Based on the Asian consensus report on FD [36], it has been suggested that “test and treat” strategy for *H. pylori* should be a treatment strategy for all patients in Asia; while this is applicable for high prevalence population, this is unlikely to be economical for the ethnic Malays. Instead, proton pump inhibitor is a reasonable first treatment strategy, also beneficial for the more common symptom of EPS in this population with response being assessed after 4 or 8 weeks. Early referral to psychiatrist is probably warranted in those with psychosocial alarm features.

## Conclusion

As a conclusion, using a reliable Malay-translated Rome III FD Diagnostic questionnaire and exclusion of erosive diseases and *H. pylori* infection, FD was found to be more common than previously expected among the ethnic Malays attending primary care clinic in a region having a low prevalence of *H. pylori* infection. There was a significant association between a positive married status and presence of psychosocial symptoms among ethnic Malays with FD especially among females.

## Competing interests

All authors declare that they have no competing interests.

## Authors’ contributions

NW carried out the study, involved in data analysis and manuscript preparation. NM, ND, NMN and ASBC provided the idea and involved in manuscript preparation. JS and YYL provided the idea, data analysis and manuscript preparation. All authors approved the final version prior to submission.

## Pre-publication history

The pre-publication history for this paper can be accessed here:

http://www.biomedcentral.com/1471-230X/13/84/prepub
